# External validation of a multivariable claims-based rule for predicting in-hospital mortality and 30-day post-pulmonary embolism complications

**DOI:** 10.1186/s12913-016-1855-y

**Published:** 2016-10-22

**Authors:** Craig I. Coleman, W. Frank Peacock, Gregory J. Fermann, Concetta Crivera, Erin R. Weeda, Michael Hull, Mary DuCharme, Laura Becker, Jeff R. Schein

**Affiliations:** 1 University of Connecticut School of Pharmacy, 69 North Eagleville Road, Storrs, CT 06269 USA; 2 Department of Emergency Medicine, Baylor College of Medicine, Houston, TX USA; 3 Department of Emergency Medicine, University of Cincinnati, Cincinnati, OH USA; 4 Janssen Scientific Affairs, LLC, Raritan, NJ USA; 5 Optum, Eden Prairie, MN USA

**Keywords:** Pulmonary embolism, Risk stratification, Mortality, Administrative claims

## Abstract

**Background:**

Low-risk pulmonary embolism (PE) patients may be candidates for outpatient treatment or abbreviated hospital stay. There is a need for a claims-based prediction rule that payers/hospitals can use to risk stratify PE patients. We sought to validate the In-hospital Mortality for PulmonAry embolism using Claims daTa (IMPACT) prediction rule for in-hospital and 30-day outcomes.

**Methods:**

We used the Optum Research Database from 1/2008-3/2015 and included adults hospitalized for PE (415.1x in the primary position or secondary position when accompanied by a primary code for a PE complication) and having continuous medical and prescription coverage for ≥6-months prior and 3-months post-inclusion or until death. In-hospital and 30-day mortality and 30-day complications (recurrent venous thromboembolism, rehospitalization or death) were assessed and prognostic accuracies of IMPACT with 95 % confidence intervals (CIs) were calculated.

**Results:**

In total, 47,531 PE patients were included. In-hospital and 30-day mortality occurred in 7.9 and 9.4 % of patients and 20.8 % experienced any complication within 30-days. Of the 19.5 % of patients classified as low-risk by IMPACT, 2.0 % died in-hospital, resulting in a sensitivity and specificity of 95.2 % (95 % CI, 94.4–95.8) and 20.7 % (95 % CI, 20.4–21.1). Only 1 additional low-risk patient died within 30-days of admission and 12.2 % experienced a complication, translating into a sensitivity and specificity of 95.9 % (95 % CI, 95.3–96.5) and 21.1 % (95 % CI, 20.7–21.5) for mortality and 88.5 % (95 % CI, 87.9–89.2) and 21.6 % (95 % CI, 21.2–22.0) for any complication.

**Conclusion:**

IMPACT had acceptable sensitivity for predicting in-hospital and 30-day mortality or complications and may be valuable for retrospective risk stratification of PE patients.

**Electronic supplementary material:**

The online version of this article (doi:10.1186/s12913-016-1855-y) contains supplementary material, which is available to authorized users.

## Background

Acute pulmonary embolism (PE) is the most serious clinical presentation of venous thromboembolic disease and has an incidence in United States (US) of ~112 events per 100,000 individuals [1]. PE results in a substantial economic burden, with annual healthcare costs per case ranging from $13,018 to $16,644 [2].

According to US and European PE treatment guidelines [3, 4], PE patients deemed at low-risk of experiencing early post-PE complications (including mortality) and who have adequate home circumstances should be considered candidates for treatment at home or following an abbreviated hospital admission. While clinical rules for the risk stratification of patients with PE are available [5], their implementation requires access to vital sign and laboratory data often incompletely reported or not found in claims databases.

Providing researchers, payers and hospital administrators with a tool that allows them to retrospectively estimate PE patients’ predicted early complication risk may facilitate epidemiologic research and aid them in making future resource utilization more efficient. The In-hospital Mortality for PulmonAry embolism using Claims daTa (IMPACT) prediction rule was derived [6] and subsequently validated in multiple external administrative databases for this purpose [7, 8] and has shown prognostic accuracy for predicting in-hospital mortality similar to that of the PE severity index (PESI), simplified PESI (sPESI) and Hestia criteria. However, sparse data supporting IMPACT’s ability to predict 30-day post-PE mortality and other complications are available [9]. Here, we sought to externally validate IMPACT’s accuracy for predicting in-hospital and 30-day outcomes using administrative claims data contained in the Optum Research Database.

## Methods

The preparation of this research report was in accordance with the Transparent Reporting of a multivariable prediction model for Individual Prognosis Or Diagnosis (TRIPOD) statement [10].

Our analysis used claims data from the Optum Research Database spanning January 2008 through March 2015. The Optum Research Database contains de-identified claims data from commercial and Medicare Advantage health plan patients and links administrative enrollment data with medical (physician, facility) and pharmacy claims [11]. Since this study utilized only de-identified patient level data via methods consistent with Health Insurance Portability and Accountability Act (HIPAA) privacy and security requirements (i.e., individual medical records or identities were not disclosed), institutional review board oversight was not required.

We included adult patients with an International Classification of Diseases, ninth-edition, Clinical Modification (ICD-9-CM) diagnosis code for PE (415.1x) in the primary position or with a secondary diagnosis code for PE along with a primary code for a PE-related complications (respiratory failure [518.81], cardiogenic shock [785.51], cardiac arrest [427.5], secondary pulmonary hypertension [416.8], syncope [780.2], thrombolysis [99.10] or intubation/mechanical ventilation [96.04, 96.05, 96.70–96.72]). Additional inclusion criteria consisted of continuous medical and prescription coverage for ≥6-months prior and 3-months post-study discharge or until death. Patients transferred from another healthcare facility were excluded as determination of low-risk status by clinicians is typically performed at the time of initial PE assessment. In addition, including transfer patients would have biased our estimates of hospital length-of-stay.

We used the IMPACT prediction rule [estimated percent absolute risk = 1/(1 + exp(−x)); where x = −5.833 + (0.026 × age) + (0.402 × myocardial infarction) + (0.368 × chronic lung disease) + (0.464 × stroke) + (0.638 × prior major bleeding) + (0.298 × atrial fibrillation) + (1.061 × cognitive impairment) + (0.554 × heart failure) + (0.364 × renal failure) + (0.484 × liver disease) + (0.523 × coagulopathy) + (1.068 × cancer)] to estimate PE patients’ risk for early all-cause mortality [6]. This claims-based logistic regression prediction tool was initially derived and internally validated in a large US MarketScan commercial and Medicare claims database by randomly assigning PE admissions between April 2010 and September 2013 into derivation (80 %) and validation (20 %) cohorts. In both cohorts, the model classified PE patients into low- and high-risk in-hospital all-cause mortality categories with high sensitivity (87 %) and moderate specificity (47 %). Consistent with prior studies, patients with an IMPACT predicted mortality risk ≤1.5 % were classified as low-risk for early mortality or other complications in our analysis [6–9]. ICD-9-CM coding for all IMPACT co-morbidities was performed according to the original IMPACT derivation paper [6]. Whenever possible, these co-morbidities were determined using Agency for Healthcare Research and Quality (AHRQ) 29-comorbidity schemas [12]; however, prior major bleeding, cognitive dysfunction, stroke, myocardial infarction and atrial fibrillation are not included in the AHRQ 29- comorbidity score and were thus identified using the procedural and diagnostic codes as listed in Additional file 1. Age was determined at time of presentation.

All-cause in-hospital and 30-day mortality as well as 30-day incidence of post-PE complications (recurrent venous thromboembolism, rehospitalization or death) served as *a priori* endpoints for this study. In-hospital and 30-day mortality were determined using the discharge status field within the claims records and from using the Social Security Administration Death Master File. Rehospitalization was said to occur if a patient had a new all-cause inpatient claim anytime following discharge for the index PE event through 30-days post-admission for the index event. Recurrent venous thromboembolism was defined as a diagnosis code for PE or deep vein thrombosis (see Additional file 2) on an emergency department or inpatient claim within 30-days of the index event. Accordingly, patients not discharged within 30-days of the index admission were not included in measures of 30-day post-admission rehospitalization and recurrent venous thromboembolism.

All baseline variables and endpoints were analyzed descriptively. Counts and percentages were provided for dichotomous or categorical variables. Means ± standard deviations or medians with 25 %, 75 % ranges were provided for continuous variables (where appropriate). To quantify the accuracy of IMPACT for predicting in-hospital and 30-day mortality as well as 30-day post-PE complications, we calculated sensitivity (the percentage of patients at high risk for a complication who are correctly identified as being high risk as evidenced by a complication occurring), specificity (the percentage of patients at low-risk of a complication who are correctly identified as being low-risk as evidenced by not experiencing a complication), positive predictive value (PPV; the probability that in the case of being classified as high-risk for a complication, the patient experiences a complication) and negative predictive value (NPV; the probability that in the case of being classified as low-risk for a complication, the patient does not experience one) along with 95 % confidence intervals (CIs). Area under-the-curve (AUC) statistics were calculated to assess the IMPACT rule’s discriminative power to correctly predict complication occurrence. All data management and statistical analysis was performed using SAS version 9.4 (SAS Institute Inc, Cary, North Carolina, USA).

## Results

In total, 47,531 PE patients were identified (Table 1). The mean patient age was 67.1 ± 15.0 years, with 63.2 % of patients ≥65 years-of-age. A majority of patients (63.3 %) were enrolled in Medicare Advantage plans rather than commercial insurance. The most common IMPACT co-morbidities observed were chronic lung disease (34.2 %), heart failure (23.1 %), atrial fibrillation (16.4 %) and cancer (15.3 %). High-risk patients were considerably older (mean age 72.1 versus 46.6 years, *p* < 0.001) and had a higher prevalence of total comorbidities than the low-risk patients. The mean IMPACT score was estimated to be 6.1 % ± 8.1 % for the total population, and differed significantly between patients at low-risk (1.1 % ± 0.3 %) or high-risk (7.3 % ± 8.6 %) for early mortality or complications (*p* < 0.001).

**Table 1:** Baseline characteristics for low- and high-risk patients

Characteristic	Total Cohort	IMPACT Low-Risk	IMPACT High-Risk
	*N* = 47,531	*N* = 9,259	*N* = 38,272
	*n* (%)	*n* (%)	*n* (%)
Age, years (mean ± SD)	67.1 ± 15.0	46.6 ± 11.1	72.1 ± 11.0
Myocardial infarction	1,647 (3.5)	14 (0.2)	1,633 (4.3)
Chronic lung disease	16,242 (34.2)	636 (6.9)	15,606 (40.8)
Stroke	2,177 (4.6)	15 (0.2)	2,162 (5.6)
Prior major bleeding	6,233 (13.1)	101 (1.1)	6,132 (16.0)
Atrial fibrillation	7,810 (16.4)	51 (0.6)	7,759 (20.3)
Cognitive dysfunction	6,489 (13.7)	3 (<0.1)	6,486 (16.9)
Heart failure	10,967 (23.1)	50 (0.5)	10,917 (28.5)
Renal failure	6,941 (14.6)	60 (0.7)	6,881 (18.0)
Liver disease	1,443 (3.0)	38 (0.4)	1,405 (3.7)
Coagulopathy	2,828 (6.0)	75 (0.8)	2,753 (7.2)
Cancer	7,287 (15.3)	4 (<0.1)	7,283 (19.0)

*SD* standard deviation

In-hospital and 30-day mortality occurred in 7.9 and 9.4 % of patients. The observed mortality risk for patients increased as estimated IMPACT mortality risk increased (see Additional file 3). A total of 20.8 % of patients experienced any post-PE complication within 30-days of the index admission. Two-by-two cross-tables of events for each endpoint are provided in Table 2. Of the 19.5 % of patients classified as low-risk by IMPACT, 2.0 % died in-hospital (versus 9.4 % in the high risk group, *p* < 0.001), resulting in a sensitivity and specificity of 95.2 and 20.7 % and a NPV of 98.0 % (Table 3). Only 1 additional low-risk patient died within 30-days of admission and 12.2 % experienced a complication, translating into a sensitivity, specificity and NPV of 95.9, 21.1 and 98.0 % for mortality and 88.5, 21.6 and 87.8 % for any complication. IMPACT’s AUCs for the in-hospital mortality, 30-day mortality and 30-day complication endpoints were 0.66, 0.68 and 0.62, respectively.

**Table 2:** 2 × 2 Tables for All-Cause In-Hospital and 30-Day Mortality and 30-Day Complications

	No All-Cause In-Hospital Death	All-Cause In-Hospital Death
IMPACT High-Risk	34,687	3,585
IMPACT Low-Risk^a^	9,077	182
	No 30-Day All-Cause Death	30-Day All-Cause Death
IMPACT High-Risk	34,013	4,259
IMPACT Low-Risk^a^	9,076	183
	No 30-Day Recurrent VTE, Rehospitalization or Death From Any Cause	30-Day Recurrent VTE, Rehospitalization or Death From Any Cause
IMPACT High-Risk	29,531	8,741
IMPACT Low-Risk^a^	8,130	1,129

*IMPACT* In-hospital Mortality for PulmonAry embolism using Claims daTa, *VTE* venous thromboembolism

^a^Low-risk defined as an “In-hospital Mortality for PulmonAry embolism using Claims daTa” rule estimated risk of early complications of ≤1.5 %

**Table 3:** Prognostic Test Characteristics for the “In-hospital Mortality for PulmonAry embolism using Claims daTa” Rule

	Impact		Complication		TP (%)	TN (%)	FP (%)	FN (%)	Sensitivity	Specificity	NPV	PPV	AUC
	Low-risk	High-risk	No	Yes					(TP/TP + FN)	(TN/TN + FP)	(TN/TN + FN)	(TP/TP + FP)	(95 % CI)
	*N*	*N*	*N*	*N*					(%, 95 % CI)	(%, 95 % CI)	(%, 95 % CI)	(%, 95 % CI)	
All-Cause In-Hospital Mortality	9,259	38,272	43,764	3,762	9.4	98.0	90.6	2.0	95.2 (94.4–95.8)	20.7 (20.4–21.1)	98.0 (97.7–98.3)	9.4 (9.1–9.7)	0.66 (0.65–0.67)
All-Cause 30-Day Mortality	9,259	38,272	43,089	4,442	11.1	98.0	88.9	2.0	95.9 (95.3–96.5)	21.1 (20.7–21.5)	98.0 (97.7–98.3)	11.1 (10.8–11.5)	0.68 (0.67–0.69)
30-Day Recurrent VTE, Rehospitalization or Death From Any Cause	9,259	38,272	37,661	9,870	22.8	87.8	77.1	12.2	88.5 (87.9–89.2)	21.6 (21.2–22.0)	87.8 (87.1–88.5)	22.8 (22.4–23.3)	0.62 (0.62–0.63)

*AUC* area under the curve statistic, *CI* confidence interval, *FN* false negative, *FP* false positive, *IMPACT* In-hospital Mortality for PulmonAry embolism using Claims daTa, *N* number, *NPV* negative predictive value, *PPV* positive predictive value, *TN* true negative, *TP* true positive, *VTE* venous thromboembolism

Median hospital length-of-stay was 4-days (25 %, 75 % range: 2–6 days) in the low-risk group and 5-days (25 %, 75 % range: 3–9 days) in the high-risk group. The percentage of patients with inpatient stays ≤2-days was 32.1 % (2975 of 9259) in the low-risk group and 20.5 % (7853 of 38,272) in the high-risk group (*p* < 0.001).

## Discussion

The multivariable IMPACT prediction rule appeared valid when applied retrospectively to the Optum Research Database. In this and prior external validation studies [6–9], IMPACT has exhibited sensitivity >90 % and NPVs ≥98 % for all-cause in-hospital mortality, but often with lower and variable specificity. While a prognostic rule would ideally be 100 % sensitive and 100 % specific; this is rarely seen in real-world scenarios. However because first and foremost clinicians aim to avoid doing harm to their patients, high sensitivity is clearly preferable (and low specificity is less important) when assessing whether a PE patient could have (claims-based tools) or should have (clinical tools) been considered for management at home or following an abbreviated hospital stay (e.g., observation status).

In this study, we also assessed 30-day all-cause mortality, and found IMPACT to have similar sensitivity and NPV (95.9 and 98.0 %, respectively) to clinical tools such as PESI, sPESI and the Hestia criteria [5]. These results support those of a smaller (*N* = 807) single-site study of computed tomography-confirmed PE patients published by Weeda and colleagues [9] that compared IMPACT’s predictive accuracy for 30-day all-cause mortality to that of PESI, sPESI and Hestia. In the study by Weeda et al. [9], IMPACT demonstrated comparable sensitivity and NPV for all-cause 30-day mortality (sensitivity = 97.0 %; NPV = 99.4 %) compared to PESI, sPESI and Hestia (30-day mortality sensitivity range = 90.9–100 % and NPV range = 98.6–100 %). This similar sensitivity is not surprising as IMPACT contains variables (including age, cancer, altered mental status, heart failure and chronic lung disease) found in commonly cited clinical rules (e.g., sPESI and/or PESI). Further, the magnitude/weight assigned to these overlapping predictors is similar across IMAPCT and clinical tools (e.g., altered mental status and cancer are heavily weighted in IMPACT and PESI). Unfortunately, we could not directly compare the prognostic accuracy of IMPACT to that of clinical rules in our analysis. While the Optum Research Database does have an electronic health record component allowing them to link vital signs and laboratory results to a proportion of covered patients, initial *a priori* pilot analyses performed in preparation for this study suggested too limited data were available to support the measurement of PESI, sPESI or the Hestia criteria. For this reason, and because IMPACT was specifically designed to risk stratify PE patients retrospectively using administrative claims data, we strongly encourage it not be used to make individual patient treatment decisions. Instead, we believe the value of IMPACT lies in its ability to retrospectively identify low-risk PE patients, making it a excellent tool for payers and hospital administrators to quickly and inexpensively benchmark rates of low-risk PE patients treated at home or following an abbreviated admission.

Of note, this study was the first to evaluate the accuracy of IMPACT for stratifying patient risk for developing a complication (recurrent venous thromboembolism, rehospitalization or death) within 30-days. IMPACT’s sensitivity for this endpoint was found to be 88.5 %; somewhat lower than observed for either in-hospital or 30-day mortality, but still likely acceptable for many decision-makers. This unique data demonstrating IMPACT’s prognostic accuracy for 30-day post-PE complications is important as various payors such as the Centers for Medicare and Medicaid Services (CMS) continue to put pressure on providers to reduced the rate of hospital readmissions [13].

Our study has other limitations worth discussing. First, while claims data are extremely valuable for the efficient and effective examination of real-world healthcare outcomes, treatment patterns, healthcare resource utilization and costs, all claims databases have inherent limitations affecting their internal and external validity. Results are dependent on the accuracy and completeness of administrative claims data, which by nature, may be prone to coding errors or omissions (for example, out of hospital mortality may have been missed due to poor reporting to the Social Security Administration and the need to link this data to Optum claims). Moreover, some limitation in the generalizability of results should always be considered because claims data are collected for the purpose of payment and not research. Second, we could not determine if the observed mortality or complications were attributable to the index PE, as this requires a prospective design and access to detailed chart data. It is likely some deaths or rehospitalizations were associated with co-morbidities (i.e., cancer and heart failure) and not directly related to the index PE. While it is unclear what influence this had on the sensitivity and specificity of IMPACT on our mortality and any complication endpoints, it may at least partially explain IMPACT’s reduced sensitivity and NPV for the any complication (30-day recurrent VTE, rehospitalization or death from any cause) endpoint. A final limitation includes the fact that claims data cannot provide information on severity of comorbidities (only their presence) and cannot fully address other factors that may be associated with the development of early complications (or the decision to keep patients in the hospital longer), including socioeconomic status, likely patient compliance to medical instructions and the presence of family support.

## Conclusion

The multivariable IMPACT rule appeared valid for predicting early (up to 30-day) post-PE outcomes when implemented in the Optum Research Database. IMPACT has previously been shown to exhibit sensitivity of ~90 % and NPV ~99 % for predicting in-hospital and 30-day mortality; and in addition to confirming these findings, this study also provides data on IMPACT’s ability to risk stratify patients for the development of recurrent venous thromboembolism, rehospitalization or death at 30-days. While not intended for clinical decision-making, IMPACT may be a valuable tool for retrospective analysis or benchmarking of PE patients.

## Additional files

Additional file 1: International Classification of Disease, Ninth Revision-Clinical Modification (ICD-9-CM) codes for independent predictors in the IMPACT model. (DOCX 12 kb)
Additional file 2: Diagnosis Codes for Recurrent Venous Thromboembolism. (DOCX 13 kb)
Additional file 3: Calibration Plot of Observed In-hospital and 30-Day Mortality. (DOCX 353 kb)

## Abbreviations

AHRQ: Agency for Healthcare Research and Quality
AUC: Area under-the-curve
CIs: Confidence intervals
CMS: Centers for Medicare and Medicaid Services
ICD-9-CM: International Classification of Diseases, ninth-edition, Clinical Modification
IMPACT: In-hospital Mortality for PulmonAry embolism using Claims daTa
NPV: Negative predictive value
PE: Pulmonary embolism
PESI: PE severity index
PPV: Positive predictive value
sPESI: simplified PESI
TRIPOD: Transparent Reporting of a multivariable prediction model for Individual Prognosis Or Diagnosis
US: United States
