# Supplementary material for: External validation of a multivariable claims-based rule for predicting in-hospital mortality and 30-day post-pulmonary embolism complications
Source: BMC Health Serv Res. 2016 Oct 22;16:610. doi: 10.1186/s12913-016-1855-y (PMC5075157; doi:10.1186/s12913-016-1855-y)
Supplement: Additional file 1: — International Classification of Disease, Ninth Revision-Clinical Modification (ICD-9-CM) codes for independent predictors in the IMPACT model. (DOCX 12 kb) [file 12913_2016_1855_MOESM1_ESM.docx]

**ADDITIONAL FILE 1. International Classification of Disease, Ninth Revision-Clinical Modification (ICD-9-CM) codes for independent predictors in the IMPACT model**

**Myocardial infarction**: 410, 412

**Chronic lung disease:** 491.x, 491.2 - 491.2, 492.0 - 492.8, 493.2x, 494, 495.x - 496.x, 516.x, 517.1, 517.2, 517.8, 518.83, 518.84

**Cerebrovascular disease:** 433.01, 433.11, 433.21, 433.31, 433.81, 433.91, 434.01, 434.11, 434.91, 437.1, 437.3

**Major bleeding:** 430, 431, 432.0, 432.1, 432.9, 852.0, 852.2, 852.4, 853.0, 455.2, 455.5, 455.8, 456.0, 456.20, 459.0, 530.7, 530.82, 531.00-01, 531.20-21, 531.40-41, 531.60-61, 532.00-01, 532.20-21, 532.40-41, 532.60-61, 533.00-01, 533.20-21, 533.40-41, 533.60-61, 534.00-01, 534.20-21, 534.40-41, 534.60-61, 535.01, 535.11, 535.21, 535.31, 535.41, 535.51, 535.61, 537.83, 562.02, 562.03, 562.12, 562.13, 568.81, 569.3, 569.85, 578.0, 578.1, 578.9, 423.0, 593.81, 599.7, 719.11, 784.7, 784.8, 786.3

**Atrial fibrillation:** 427.31

**Cognitive impairment:** 290, 294.10, 294.11, 294.2, 294.8, 331.0, 331.1, 331.11, 331.19, 331.2, 331.82, 331.83, 438.0, 780.01, 780.02, 780.09, 780.93, 780.97, 797, 907.0

**Heart failure:** 398.91, 402.01, 402.11, 402.91, 404.01, 404.11, 404.91, 428.0-428.9

**Renal failure:** 403.01, 403.11, 403.91, 404.02, 404.03, 404.12, 404.13, 404.92, 404.93, 585, 586, V42.0, V45.1, V56.0-V56.2, V56.8

**Liver disease:** 070.22, 070.23, 070.32, 070.33, 070.44, 070.54, 456.0, 456.1, 456.20, 456.21, 571.0, 571.2, 571.3, 571.40-571.49, 571.5, 571.6, 571.8, 571.9, 572.3, 572.8, V42.7

**Coagulopathy:** 286.0-286.9, 287.1, 287.3-287.5, 289.81-289.82

**Cancer:** 200.00-202.38, 202.50-203.01, 203.8-203.81, 238.6, 273.3, V10.71, V10.72, V10.79, 196.0-199.1, 140.0-172.9, 174.0-175.9, 179-195.8, V10.00-V10.59, V10.81-V10.9
