# Supplementary material for: External validation of a multivariable claims-based rule for predicting in-hospital mortality and 30-day post-pulmonary embolism complications
Source: BMC Health Serv Res. 2016 Oct 22;16:610. doi: 10.1186/s12913-016-1855-y (PMC5075157; doi:10.1186/s12913-016-1855-y)
Supplement: Additional file 2: — Diagnosis Codes for Recurrent Venous Thromboembolism. (DOCX 13 kb) [file 12913_2016_1855_MOESM2_ESM.docx]

**ADDITIONAL FILE 2. Diagnosis Codes for Recurrent Venous Thromboembolism**

| **VTE Type** | **Code** | **Code Type** | **Full Description** |
| --- | --- | --- | --- |
| DVT | 451.11 | ICD-9 Dx | Phlebitis and thrombophlebitis of femoral vein (deep) (superficial) |
|  | 451.19 |  | Phlebitis and thrombophlebitis of other deep vessels of lower extremities |
|  | 451.2 |  | Phlebitis and thrombophlebitis of lower extremities, unspecified |
|  | 451.81 |  | Phlebitis and thrombophlebitis of iliac vein |
|  | 451.82 |  | Phlebitis and thrombophlebitis of superficial veins of upper extremities |
|  | 451.83 |  | Phlebitis and thrombophlebitis of deep veins of upper extremities |
|  | 451.84 |  | Phlebitis and thrombophlebitis of upper extremities, unspecified |
|  | 451.89 |  | Phlebitis and thrombophlebitis of other site |
|  | 451.9 |  | Phlebitis and thrombophlebitis of unspecified site |
|  | 452 |  | Portal vein thrombosis |
|  | 453.1 |  | Thrombophlebitis migrans |
|  | 453.2 |  | Other venous embolism and thrombosis, of inferior vena cava |
|  | 453.3 |  | Embolism and thrombosis of renal vein |
|  | 453.40 |  | Acute venous embolism and thrombosis of unspecified deep vessels of lower extremity |
|  | 453.41 |  | Acute venous embolism and thrombosis of deep vessels of proximal lower extremity |
|  | 453.42 |  | Acute venous embolism and thrombosis of deep vessels of distal lower extremity |
|  | 453.50 |  | Chronic venous embolism and thrombosis of unspecified deep vessels of lower extremity |
|  | 453.51 |  | Chronic venous embolism and thrombosis of deep vessels of proximal lower extremity |
|  | 453.52 |  | Chronic venous embolism and thrombosis of deep vessels of distal lower extremity |
|  | 453.6 |  | Venous embolism and thrombosis of superficial vessels of lower extremity |
|  | 453.71 |  | Chronic venous embolism and thrombosis of superficial veins of upper extremity |
|  | 453.72 |  | Chronic venous embolism and thrombosis of deep veins of upper extremity |
|  | 453.73 |  | Chronic venous embolism and thrombosis of upper extremity, unspecified |
|  | 453.74 |  | Chronic venous embolism and thrombosis of axillary veins |
|  | 453.75 |  | Chronic venous embolism and thrombosis of subclavian veins |
|  | 453.76 |  | Chronic venous embolism and thrombosis of internal jugular veins |
|  | 453.77 |  | Chronic venous embolism and thrombosis of other thoracic veins |
|  | 453.79 |  | Chronic venous embolism and thrombosis of other specified veins |
|  | 453.81 |  | Acute venous embolism and thrombosis of superficial veins of upper extremity |
|  | 453.82 |  | Acute venous embolism and thrombosis of deep veins of upper extremity |
|  | 453.83 |  | Acute venous embolism and thrombosis of upper extremity, unspecified |
|  | 453.84 |  | Acute venous embolism and thrombosis of axillary veins |
|  | 453.85 |  | Acute venous embolism and thrombosis of subclavian veins |
|  | 453.86 |  | Acute venous embolism and thrombosis of internal jugular veins |
|  | 453.87 |  | Acute venous embolism and thrombosis of other thoracic veins |
|  | 453.89 |  | Acute venous embolism and thrombosis of other specified veins |
|  | 453.9 |  | Embolism and thrombosis of unspecified site |
|  | 671.30 |  | Deep phlebothrombosis, antepartum, unspecified as to episode of care |
|  | 671.31 |  | Deep phlebothrombosis, antepartum, with delivery |
|  | 671.33 |  | Deep phlebothrombosis, antepartum |
|  | 671.40 |  | Deep phlebothrombosis, postpartum, unspecified as to episode of care |
|  | 671.42 |  | Deep phlebothrombosis, postpartum, with delivery |
|  | 671.44 |  | Deep phlebothrombosis, postpartum |
|  | 671.50 |  | Other phlebitis and thrombosis complicating pregnancy and the puerperium, unspecified as to episode of care |
|  | 671.51 |  | Other phlebitis and thrombosis with delivery, with or without mention of antepartum condition |
|  | 671.52 |  | Other phlebitis and thrombosis with delivery, with mention of postpartum complication |
|  | 671.53 |  | Other antepartum phlebitis and thrombosis |
| PE | 415.11 | ICD-9 Dx | Iatrogenic pulmonary embolism and infarction |
|  | 415.12 |  | Septic pulmonary embolism |
|  | 415.13 |  | Saddle embolus of pulmonary artery |
|  | 415.19 |  | Other pulmonary embolism and infarction |
|  | 416.2 |  | Chronic pulmonary embolism |
|  | 416.8 |  | Secondary pulmonary embolism |
|  | 673.20-673.24 |  | Obstetrical blood-clot embolism |
|  | 673.80 |  | Other obstetrical pulmonary embolism, unspecified as to episode of care |
|  | 673.81 |  | Other obstetrical pulmonary embolism, with delivery, with or without mention of antepartum condition |
|  | 673.82 |  | Other obstetrical pulmonary embolism, with delivery, with mention of postpartum complication |
|  | 673.83 |  | Other obstetrical pulmonary embolism, antepartum |
|  | 673.84 |  | Other obstetrical pulmonary embolism, postpartum |
| PE | 38.7 | ICD-9 Proc | Interruption of the vena cava |
