# Supplementary material for: External validation of a multivariable claims-based rule for predicting in-hospital mortality and 30-day post-pulmonary embolism complications
Source: BMC Health Serv Res. 2016 Oct 22;16:610. doi: 10.1186/s12913-016-1855-y (PMC5075157; doi:10.1186/s12913-016-1855-y)
Supplement: Additional file 3: — Calibration Plot of Observed In-hospital and 30-Day Mortality. (DOCX 353 kb) [file 12913_2016_1855_MOESM3_ESM.docx]

**ADDITIONAL FILE 3. Calibration Plot of Observed In-hospital and 30-Day Mortality**


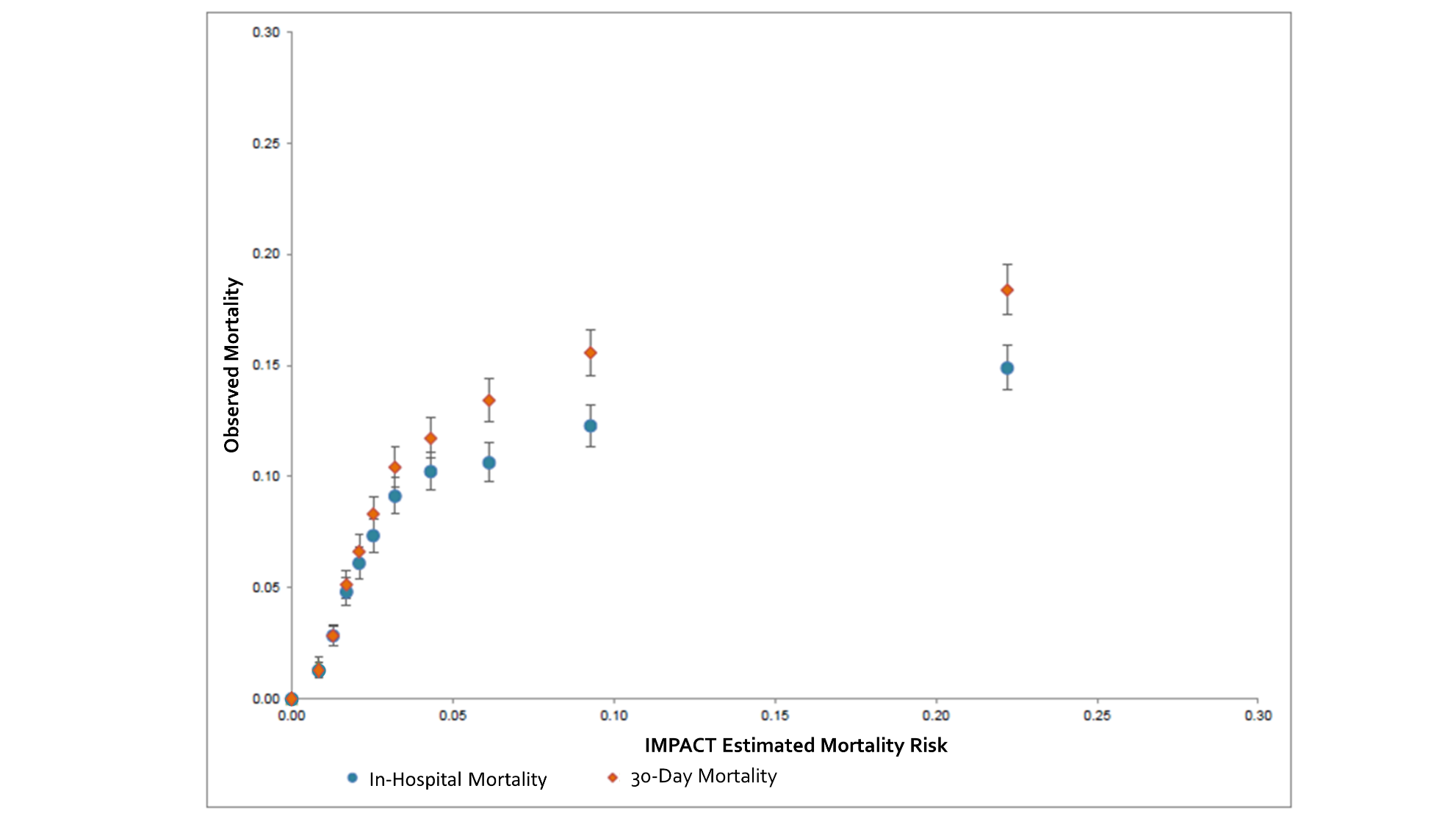


IMPACT= In-hospital Mortality for PulmonAry embolism using Claims daTa
